# Changes in Men’s Salivary Testosterone and Cortisol Levels, and in Sexual Desire after Smelling Female Axillary and Vulvar Scents

**DOI:** 10.3389/fendo.2013.00159

**Published:** 2013-10-28

**Authors:** Ana Lilia Cerda-Molina, Leonor Hernández-López, Claudio E. de la O, Roberto Chavira-Ramírez, Ricardo Mondragón-Ceballos

**Affiliations:** ^1^Departamento de Etología, Instituto Nacional de Psiquiatría Ramón de la Fuente Muñiz, Ciudad de México, México; ^2^Departamento de Biología de la Reproducción, Instituto Nacional de Ciencias Médicas y la Nutrición Salvador Zubirán, Ciudad de México, México

**Keywords:** semiochemical communication, women’s attractivity, menstrual cycle, sexual desire, testosterone, cortisol

## Abstract

Several studies have shown that a woman’s vaginal or axillary odors convey information on her attractivity. Yet, whether such scents induce psychoneuroendocrinological changes in perceivers is still controversial. We studied if smelling axillary and vulvar odors collected in the periovulatory and late luteal phases of young women modify salivary testosterone and cortisol levels, as well as sexual desire in men. Forty-five women and 115 men, all of them college students and unacquainted with each other, participated in the study. Female odors were collected on pads affixed to the axilla and on panty protectors both worn the entire night before experiments. Men provided five saliva samples, a basal one before the smelling procedure, and four more 15, 30, 60, and 75 min after exposure to odors. Immediately after smelling the odor source, men answered a questionnaire rating hedonic qualities of scents, and after providing the last saliva sample they answered questionnaire on sexual desire. We found that periovulatory axillary and vulvar odors increased testosterone and cortisol levels, with vulvar scents producing a more prolonged effect. Luteal axilla odors decreased testosterone and cortisol levels, while luteal vulva odors increased cortisol. Periovulatory axilla and vulva scents accounted for a general increase of interest in sex. These odors were also rated as more pleasant and familiar, while luteal vulvar odors were perceived as intense and unpleasant.

## Introduction

Beach (1) defined *attractivity* as “female’s stimulus value in evoking sexual responses by the male.” Recent studies suggest that men are able to detect female attractivity by odor. Torso and axillary odors of women in the follicular phase are rated as significantly more attractive than those of women in the luteal phase (2–5). Miller and Maner (6) found that men’s salivary testosterone increases after smelling T-shirts worn three consecutive nights by women near ovulation, but decreases if these clothes were used during the luteal phase. However, two independent studies have failed to replicate these last findings (7, 8), and differences in the experimental designs regarding men’s awareness of what they were smelling, the time taken to collect odors, or whether odors belonged to known or strange women, to name a few, prevent the comparison of results. Therefore, although human steroid metabolites account for pheromonal-like effects (9), provide relevant cues for mate choice (9, 10) and signal ovulation (see above), whether female scents increase male sexual arousal by way of neuroendocrine pathways is debatable.

Certain non-human male primates use urinary (11) or vaginal and perineum scents (12, 13) to gather information about females’ reproductive condition. Testosterone increases [following a luteinizing hormone (LH) surge] in male stump-tailed macaques exposed to the vaginal odors produced around the time of ovulation (14). Concerning human genitals, Michael et al. (15), and more recently Levin (16) proposed that the role of female vaginal secretions in semiochemical communication is to explicitly induce sexual arousal.

The vulvar area, extending from the *mons pubis* to the *perineum*, is rich in exocrine glands such as the Bartholin’s and Skene’s glands, while the sebaceous glands of the *labia majora* is the exit of vaginal secretions (17) and the place of the more recently discovered anogenital “sweat” glands (18). Yet, studies concerning the role of female genital scents have been largely confined to vaginal secretions [review in Ref. (19)]. For example, men and women perceive vaginal ovulatory secretions as less unpleasant and much less intense than secretions produced on the other days of the menstrual cycle (20). Vaginal secretions contain around 2100 odoriferous compounds, of which 34 are related to hedonic sensations while smelling it (21). However, the presence of specialized exocrine glands in the vulvar area, along with the fact that human chemical communication might involve mixtures rather than isolated compounds (22), suggests that the secretions of these glands, together with vaginal secretions and the normal metabolism of bacterial flora, would contribute to the formation of semiochemicals. Skene’s glands seem responsible for female emission or ejaculation during orgasm (23), but this does not exclude that these glands can secrete at other times. Berman (24) mentions (without citing) that some authors claim that human Bartholin’s gland secretions emit an odoriferous fluid to attract males. In female hamsters, the protein aphrodisin, a pheromone produced by the Bartholin’s glands facilitates male copulatory behavior (25).

Considering that full bipedalism appeared around two million years ago, with *Homo ergaster* (26), historically followed by clothing, and at sometime intimacy to copulate, a question of interest is why the human female genitals should retain semiochemical functions adequate to quadruped mammals. Taking this in account, intimate situations (at least in historic and modern populations) are needed so that men comfortably smell the female genitals. Cunnilingus is a suitable sexual practice to gather semiochemical information of scents and flavors found in the female genitalia (interestingly, for the armpit there is a similar practice: maschalagnia (27). Cunnilingus has attracted the interest of evolutionary psychologists, who have hypothesized that if there is a risk of cuckoldry (1) it serves to detect semen odor in their partner’s genitals and to counteract the effects of a rival’s previous ejaculation (28); (2) to promote sperm retention orgasms (29), or (3) as a mate retention behavior by increasing partner’s relationship satisfaction that promotes future copulations (30). Yet, to our knowledge, no attention has been given to the likelihood that olfactory stimulation occurring during cunnilingus is also rewarding to the male, e.g., acting as a releaser semiochemical that could enhance men’s sexual stamina. For example, pre-copulatory cunnilingus increases the duration of copulation in Indian flying foxes, *Pteropus giganteus* (31).

Since similar exocrine glands are found in the axilla and the vulva, we hypothesized that both body parts scents can inform about females’ attractivity and affect current interest in having sex. For that, we compared vulva and axilla scents collected in the periovulatory phase with scents collected in the luteal phase. Increases in testosterone levels following mild intra-sexual competition in men also promote affiliative behaviors with women (32). Sexually arousing stimuli, such as viewing erotic films, increase men’s LH and testosterone (33, 34). Yet, sexually arousing stimuli affect other hormones, including a decrease of cortisol (35, 36). On the other hand, cortisol increases in squirrel monkeys when males are housed with new females (37), and also in young men when socially interacting with unknown women (38, 39). It is known that cortisol increases in humans in response to events threatening to self-esteem (40), a situation likely to arise upon meeting a potential new sex partner. Thus, we expected that if women’s odors have some effect upon sexual arousal, testosterone, cortisol, and interest in sex would increase after smelling periovulatory scents, whether axillary or vulvar, as a proxy for readiness to meet and interact with a potential sexual partner (38). In addition, we expected that participants would rate such odors as highly hedonic. On the other hand, we expected a decrease in testosterone levels and current interest in sex after smelling luteal odors, besides from rating these as disgusting, all these signaling sexual disinterest. However, we did not expect changes in cortisol levels, since sexual disinterest seems unlikely to elicit anticipatory cognitive appraisal.

## Materials and Methods

### Participants

Forty-five women (age: mean ± SD = 22 ± 2.8 years) and 115 men (23 ± 5.5 years) participated in the study. All were college students recruited by posters asking for male and female volunteers willing to participate in a research on perception of body odors to present at the Ethology Department of the Instituto Nacional de Psiquiatría Ramón de la Fuente Muñiz (INPRFM). Participants belonged to the Nursing, Sciences, Psychology, and Engineer Faculties of the Universidad Nacional Autónoma de México and the Escuela Nacional de Antropología e Historia in Mexico City. Female researchers interviewed women volunteers and male researchers interviewed men volunteers. Women were asked to fill a brief questionnaire answering if they knew how long was their menstrual cycle; if they had had any irregular cycles in the past 6 months; if they kept track of the cycle; if they took hormonal contraceptives; if they were currently in a long-term relationship (dating or living with a partner for more than 6 months); what was their sexual orientation; if they have or had suffered any sort of genital infection (e.g., bacterial, candidiasis, trichomoniasis) in the past 3 months; if they smoked. The 45 women chosen to participate in the study were all heterosexual, had regular cycles of 28–30 days, kept calendar track of the cycle, were healthy and had not suffered any gynecological illness, were not taking hormonal contraceptives and did not smoke. Once accepted as a participant, each woman was told to wait until the beginning of her next menstrual cycle and to present to the laboratory 1 or 2 days before reaching midcycle, and 2 or 3 days before she thought would start menstruating.

Male participants also filled a questionnaire answering what was their sexual orientation; if they were in a long-term relationship (same as women); if they took anabolic steroids; if they were heavy drinkers or recreational drug users; if they smoked. The 115 men we recruited were all heterosexual, non-smokers, not heavy drinkers, not drug users, and not taking anabolic steroids. Once accepted, they were asked to provide their cell phone number, house telephone number, and e-mail address in order to let them know in advance when they had to present to the laboratory. None of the men volunteers came from the same female participants’ faculties, and 59 (51%) reported to be in a long-term relationship. It is worth noting that upon arriving to the interview most of the men asked if the study was about “pheromones.” Even though the researchers’ answer was “no,” it shows that male participants had already in mind an accurate idea of the study.

### Ethics

The study adheres to the Declaration of Helsinki and the Mexican Official Norm for Research with Human Beings (NOM-012-SSA3-2012, http://dof.gob.mx/nota_detalle.php?codigo=5284148&fecha=04/01/2013) and was approved by the Bioethics Committee of the INPRFM. Since the NOM-012-SSA3-2012 requires disclosing to the volunteers the kind of research they are going to participate in, during recruitment interviews we told female volunteers their odors were going to be smelled and qualified by unknown men, while men were told they were going to breathe harmless body odors (without giving information of the scent source or gender), provide saliva samples and answer two short questionnaires. If they had no objections to collaborate, the participants signed a consent of agreement. Volunteers were paid around 15 USD for their collaboration; women were paid twice, once for each time they provided odor samples, while men were paid once for participating in the experiment. Payments were done the day they showed up to provide the samples or to the smelling test.

### Collection of body odors

When women presented to the laboratory 1 or 2 days before midcycle or before menstruation, they were given a clean, sterile cotton pad and a winged cotton panty protector wrapped separately in plastic bags, as well as one small roll of medical micropore tape. They were instructed to wear the cotton pad in the armpit overnight (affixed with micropore tape) and the panty protector (taking care it covered all the vulvar zone) for at least 8 h during the middle day of their menstrual cycle, and during the night 24 h before menstruation. In this way we tried to minimize the bacterial flora breakdown of fresh odorless secretions into foul odorous compounds (41). Women were also required to abstain from sexual intercourse for 24 h before wearing and returning the garments, from taking afternoon or night showers, and from eating spicy or heavily spiced food on those days. Upon awakening, they had to repack the cotton pad and the panty protector separately in the plastic bags and return them first thing in the morning (around 08:00–09:00 h) to the female researchers. While in the lab, the women provided two 6 ml samples of saliva for further estradiol and progesterone evaluation; they were weighted and had their hips and waists circumferences measured. Estradiol and progesterone were used to confirm their menstrual cycle phase. Our expected balance design of 45 odor samples for menstrual phase per odor source became unbalanced because 13 women in the periovulatory phase did not provide the vulvar sample; 8 from the above women and 3 more did not return to provide the luteal samples, and another 8 women provided the luteal axilla but not the vulvar sample.

On the day a woman attended the lab to gather the cotton pad and panty protector, three men were contacted and asked to present to the lab at 1 or 2 days later at 09:00 h (depending on the time the female student presented herself). They were also asked not to have sexual relations for 24 h before presenting to the lab.

### Procedure

To minimize cueing the participants about the purpose of the study, we used a double-blind experimental design where participants did not know what they were smelling, while male experimenters (who attended male volunteers) were unaware of the odor condition being tested. Every two men smelled odors from a single female: one from the axilla (cotton pad) and the other from the vulva (panty protector), while a third one smelled air. The smelling experiments were done the same morning (09:00–11:00 h) on which the odor samples arrived, when cortisol and testosterone levels are high (42, 43). Female researchers placed each odor source inside the medicine compartment of a piston compressor nebulizer (Model 3142, volume: 10 cm^3^, minimal air flow: 0.33 ml/l, maximal pressure: 5 l/min; Technoneb, Argentina) before male researchers arrived with the participants. Each man was taken to a different room and sat in front of a desk on which were placed five assay tubes the nebulizer, a DVD player, and a TV set, an envelope enclosing the questionnaires and a packet of sugarless chewing gum. The use of chewing gum to stimulate saliva production was done *ad libitum*. The volunteer was asked to provide a first (basal) 6 ml saliva sample; he was then given a written set of instructions and a verbal explanation about the rest of the procedure. They had to cover their nose and mouth with the nebulizer’s mask, breathe through nose and mouth for 5 s while pressing the “On” button, release the button for 10 s, breathe again for 5 s, and so on until accomplishing 2 min (all these to minimize habituation). Immediately after, they had to answer a questionnaire about the perception of odors. Thereafter, to mitigate boredom, men were asked to watch a BBC video about whales or ocean life, providing further saliva samples 15, 30, 60, and 75 min after finishing the inhalation procedure. We chose these times to collect saliva samples knowing that an increase in men’s salivary testosterone becomes noticeable at 15 min following exposure to an erotic stimulus (33), while salivary cortisol levels peak around 10–30 min following a stressful stimulus (44). A buzzer connected to a timer notified participants it was time to provide the sample. Once they gave the last saliva sample, they had to open the envelope and answer the “interest in sex” questionnaire. Given the unbalance introduced by the missing odors samples, 25 men smelled air, 45 periovulatory axilla odors, 32 periovulatory vulva odors, 34 luteal axilla odors, and 26 luteal vulva odors.

### Interest in sex and hedonic properties of odors questionnaires

The “interest in sex” questionnaire consisted of six Likert items (0–8 scale: Cronbach’s α = 0.705): (1) you think that your sexual desire normally is? (0 = very low, 8 = extremely high); (2) would you like to have sex right now? (0 = no, 8 = absolutely); (3) if you were to have sex right now, how “hot” would you be? (0 = none at all, 8 = extremely hot); (4) compared to when you arrived, how much are you interested in having now sex? (0 = much less, 8 = much more); (5) compared to when you arrived, how much are you interested right now in indulging yourself masturbating, watching or reading porn, flirting at a bar or disco, going to a table dance club? (0 = much less, 8 = too much); (6) how long would you endure without having sex? (0 = my entire life, 8 = less than a day). For analyses we averaged each participant scores.

The hedonic properties of odors questionnaire had four questions: (1) did you smell something (yes, no); (2) how familiar are you with this odor; (3) how intense is this odor; (4) how pleasant is this odor. Answers to questions 2–4 consisted of a Likert scale (0 = none at all, 6 = very much). Participants had to answer these questions only if they answered “yes” in the first one.

### Hormone analyses

As soon as each experiment ended, saliva samples were immediately frozen in acetone and dry ice and stored at −70°C. We collected a total of 570 saliva samples. To free the samples from mucopolysaccharides and proteins they were subjected to three subsequent freeze-thaw cycles. Upon thawing samples were centrifuged at 3000 rpm × 30 min, the supernatants were collected and the samples were again frozen (45). We measured testosterone and cortisol by chemiluminescence (IMMULITE 1000, Siemens, TX, USA). Inter-assay and intra-assay coefficients for testosterone were 8.95 and 8.02% respectively. Cortisol inter-assay coefficient was 8.25% and the intra-assay coefficient was 7.79. The lower limit of sensitivity for testosterone was 0.0004 nmol/l and for cortisol 0.037 nmol/l. We did not test for cross-reactions. The manufacturer report on testosterone cross-reactions with another androgens are: androstenedione, 0.8%, 5α-androstan-3β,17β-diol, 0.4%; 5α-dihyrotestosterone, 2.4%; 5α-androstan-3,17-dione, 0%; 5-androsten-3β,17β-diol, not detectable. Cross-reactions with another glucocorticoids are: corticosterone, 8.6%, fluorocortisone, 0.2% tetrahydrocortisol, 0.9%, cortisone, not detectable. Basal testosterone (mean ± SD: 23.9 ± 6.7 nmol/l) and cortisol (8.3 ± 3 nmol/l) were in the normal ranges for the time of day (42, 43). As has been found in other studies (46, 47), men who were in a long-term relationship had significant lower basal testosterone than the remaining participants (mean ± SE: long-term: 22.85 ± 1.11 nmol/l; single: 27.3 ± 1.74 nmol/l; *t*_(107.7)_ = −2.15, *p* = 0.03, *d* = 0.41). Mean basal cortisol levels did not vary with relationship status.

### Analyses

Testosterone values were normally distributed within odor sources and time of collection of saliva samples, while ratings of familiarity and intensity were normally distributed within odor sources. Cortisol, scores of the interest in sex, and ratings of pleasantness were not normally distributed. Therefore, we did analyses on log-transformed cortisol values, and square root transformed scores of interest in sex and ratings of pleasantness. Our hormone sampling was not suitable for traditional repeated measures ANOVA, as odor sources for some women were missing and in the case of complete within-women samples, two distinct men breathed odors from a same female participant (accounting for autocorrelation). Therefore, we used linear mixed models (48) to analyze the hormonal data, with time and odor source (air, periovulatory axilla, periovulatory vulva, luteal axilla, luteal vulva), as fixed effects, and female (or air test number), male participants identities, and relationship status as random effects. Linear mixed models were also used to analyze the interest in sex questionnaire and the hedonic properties of scents, odor source being the only fixed effect, and female (or air test number), male participants identities, and relationship status the random effects. We used Dunnet’s test in *post hoc* contrasts when comparing the effects of the odor sources with air values. Following the recommendations of Dickinson et al. (49), we calculated effect sizes *d* (50) for the *post hoc* contrasts in order to support significant findings. The data were analyzed using SPSS 17 and effect sizes were obtained with G*Power 3.1 (51). All tests were two-tailed and significance was set at *p* ≤ 0.05.

## Results

### Hormones

**Figure 1:**
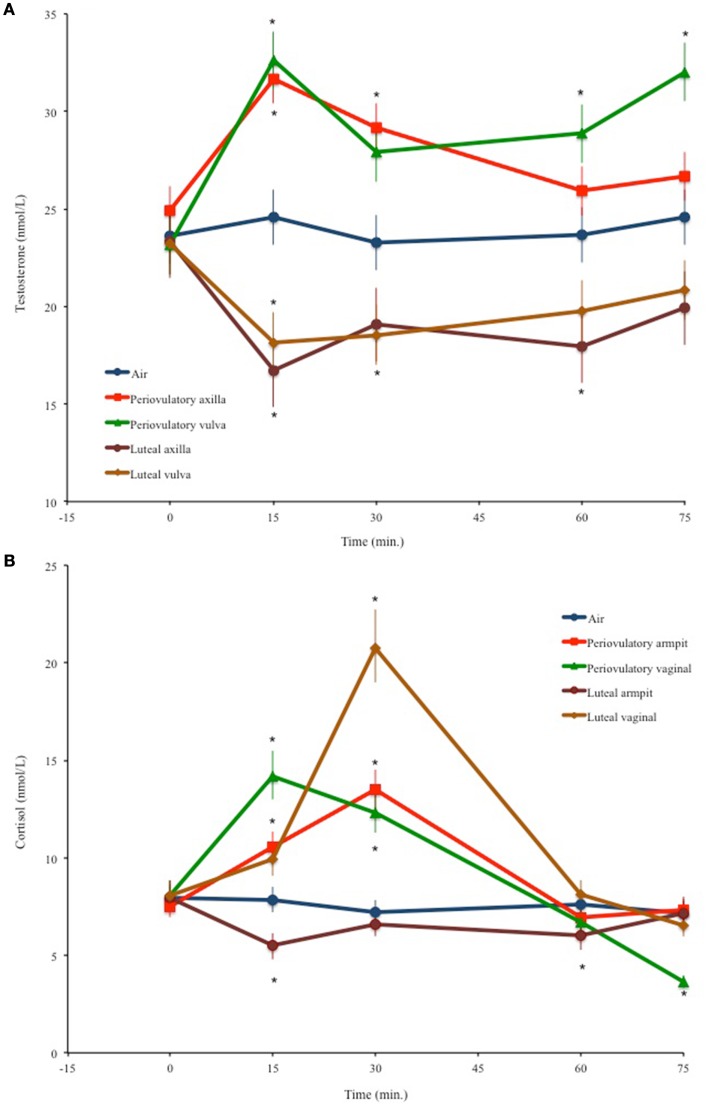
**Men’s mean (±SEM) temporal variations of (A) salivary testosterone and (B) salivary cortisol levels after smelling air or different female odors**. *p < 0.05 compared to basal values.

Testosterone values changed significantly through time depending on the odor source [*F*_(16,436)_ = 4.66, *p* < 0.0001]; Figure 1A resumes these results. Testosterone did not vary in men smelling air. Significant increases in testosterone with respect to basal values were observed at 15 (*p* < 0.0001, *d* = 0.54) and 30 min (*p* = 0.011, *d* = 0.27) after smelling periovulatory axilla odors. Smelling periovulatory vulva odors significantly increased testosterone at 15 (*p* < 0.0001, *d* = 0.61), 60 (*p* = 0.0034, *d* = 0.3), and 75 min (*p* < 0.0001, *d* = 0.57). Luteal axilla odors significantly decreased testosterone at 15 (*p* = 0.007, *d* = 0.57) and at 60 min (*p* = 0.034, *d* = 0.46), while luteal vulva odors accounted for significant decreases of testosterone at 15 (*p* = 0.003, *d* = 0.48) and 30 min (*p* = 0.007, *d* = 0.44).

The odor source × time interaction also accounted for significant variations in cortisol concentrations [*F*_(16,436)_ = 12.79, *p* < 0.0001]. Figure 1B shows the back-transformed means and standard errors for the odor source × time interaction. Smelling air had no effect on cortisol. After smelling periovulatory axilla scents, cortisol increased at 15 min (*p* = 0.001, *d* = 0.36), peaking at 30 (*p* < 0.0001, *d* = 0.63). Periovulatory vulva odors elicited a cortisol peak at 15 min (*p* < 0.0001, *d* = 0.61). By 30 min, cortisol values had decreased, but were still significantly above basal measurements (*p* = 0.001, *d* = 0.46); however, at 75 min they were significantly below the basal values (*p* < 0.0001, *d* = 0.87). Cortisol decreased significantly at 15 (*p* < 0.0001, *d* = 0.87) and 60 min (*p* < 0.0001, *d* = 0.66) after smelling luteal axilla odors. Luteal vulva odors accounted for a cortisol peak at 30 min (*p* < 0.0001, *d* = 0.96).

### Interest in sex

**Figure 2:**
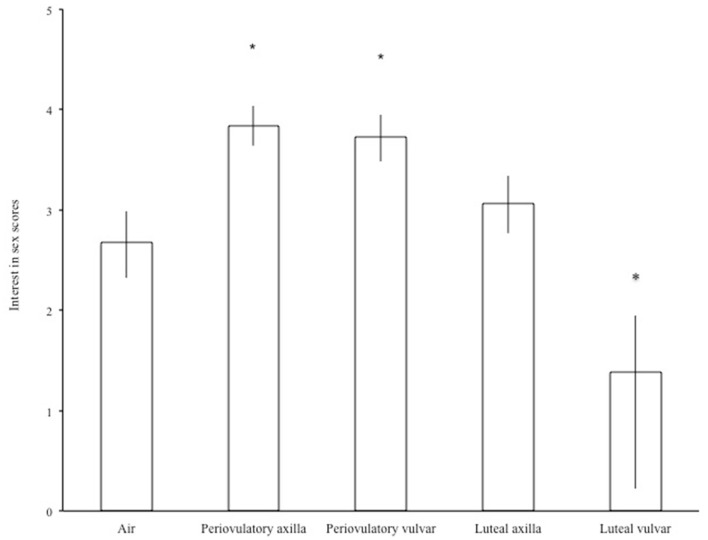
**Men’s mean (±SEM) “interest in sex” scores following the smelling of air or different female odors**. *p < 0.05 compared to air.

Odor source had significant effects on interest in sex [*F*_(4,110)_ = 21.46, *p* < 0.0001]. Figure 2 shows back-transformed interest in sex scores with respect to odor source. Both periovulatory axilla and periovulatory vulva odors significantly increased interest in sex (periovulatory axilla: *p* < 0.0001, *d* = 0.84; periovulatory vulva: *p* = 0.004, *d* = 0.4). On the other hand, odors from the luteal phase had no effect on interest in sex.

### Hedonic ratings

Seventy-eight participants reported having perceived an odor, 15 of them who smelled air. Excluding the air condition from the analyses (*n* = 25), we found no association between odor source and odor perception [χ^2^_(3)_ = 2.02, *p* = 0.6]. Significantly more participants reported having perceived something after smelling an active odor source [yes/no: 63/27; χ^2^_(1)_ = 14.4, *p* < 0.0001]. Odor source significantly affected how participants rated hedonic properties of odors [familiarity, *F*_(4,73)_ = 4.63, *p* = 0.002; intensity: *F*_(4,73)_ = 3.89, *p* = 0.006; pleasantness: *F*_(4,73)_ = 9.03, *p* < 0.0001]. Table 1 shows the mean ratings for familiarity, intensity, and pleasantness with respect to odor source. Compared with air, men perceived as significantly more familiar periovulatory axilla (*p* = 0.021, *d* = 0.44), periovulatory vulva (*p* = 0.02, *d* = 0.52), and luteal vulva (*p* = 0.001, *d* = 0.69) odors. Luteal vulva odors were rated as the most intense (*p* < 0.0001, *d* = 0.89). Men perceived periovulatory axilla (*p* = 0.002, *d* = 0.54) and periovulatory vulva (*p* = 0.008, *d* = 0.41) scents as significantly more pleasant than air, but luteal vulva odors as significantly less pleasant (*p* = 0.046, *d* = 0.34).

**Table 1:** **Hedonic ratings given to air and female odors**.

Source	Familiarity		Intensity		Pleasantness^a^	
	Mean	SE	Mean	SE	Mean	SE
Air	2.5	0.3	2.1	0.3	2.7	1.3
Periovulatory axilla	3.5*	0.3	2.6	0.3	3.8*	1.2
Periovulatory vulva	3.5*	0.3	2.6	0.3	3.7*	1.3
Luteal axilla	2.5	0.3	2.7	0.3	3.1	1.3
Luteal vulva	4.1*	0.3	3.8*	0.3	1.4*	1.4

*^a^ Back-transformed means and SE*.

*p < 0.05 compared with air.

## Discussion

Our results show that female odors from two distinct body parts, the axilla and the vulva, elicit hormonal changes in men that smell them according to the menstrual cycle’s phase. Yet, it should be noted that most of our male participants hinted being aware what they were going to smell (see Participants). Therefore, alike Miller’s and Maner’s (6) study, which told their participants they would smell female odors, we cannot exclude a bias introduced by sexual fantasying. The fact that 60% of the men in the smelling air condition reported having perceived an odor and rated its familiarity, intensity, and pleasantness supports this idea.

We attribute to the time cotton pads were affixed to the axilla (around 8–10 h) our results showing that axillar odors inform about women’s attractivity. Havlícec et al. (41) have shown that body odor sampling length is a crucial factor in how odor raters perceive scents. Roney and Simmons (7) used odors sampled for a very brief period following a brisk walk until female donors started sweating for 5 min before they returned the cotton pad affixed to the armpit to the researchers. From their description of the procedure it is difficult to calculate how much time in average each of their female volunteers worn the cotton pad, but seemingly it was no much more than an hour. Perhaps too short time to collect enough scents’ concentrations to elicit any kind of effect. On the other hand, Strom’s et al. (8) study, although relying in a large sample of couples, did not test experimentally the effects of smelling odors, nor considered that men in a long-term relationship have lower testosterone concentrations than single men, nor took in account habituation to partners’ scents. Yet, our own results might also be biased owing to the fact that besides smelling, scents were pumped into the participants’ nostrils and oral cavity, thus inhaling greater amount of molecules (in parts per million) than the ones brought to the nose and mouth by the sheer force of smelling. In common with all studies investigating airborne compounds, chemical communication studies done in humans are still plagued with dose-effect issues.

The endocrine results confirm that female odors signal reproductive status, inducing appropriate male physiological responses to deal with a potential mate and intra-sexual competition (32, 39). Testosterone increased after smelling periovulatory odors, but the periovulatory vulva facilitated a more prolonged effect than the periovulatory axilla. The shared effect of axillary and vaginal odors might arise from the fact that apocrine glands involved in the synthesis of putative steroidal pheromone-like compounds, such as androstanedione and estratetraenol (52, 53) are found mainly in the axilla (54), but also in the *labia majora* (17) and the *perineum* (18). The extended increase of testosterone elicited by periovulatory vulva odors suggests that genital compounds are more diverse and potent than those from the axilla, perhaps owing to the presence of more specialized glands in that area. Luteal odors decreased testosterone. As mentioned, this result was first reported by Miller and Maner (6), and might elicit a low testosterone status that decreases sexual desire (55), or at least make women in the non-fertile days sexually uninteresting.

Cortisol also changed, according to our predictions, when periovulatory odors were involved: both axilla and vulva odors significantly increased this glucocorticoid salivary level in the short term (15–30 min), in a similar way to when men meet strange women (38, 39). However, after half an hour, the effects of axilla and vulva odors were somewhat different. Periovulatory vulva odors elicited a complex cortisol response, peaking at 15 min followed by a steady decrease, which reached a nadir at 75 min. This decrease in cortisol resembles the decrease that occurs in men (35) and women (36) while watching an erotic film. Yet, since testosterone and cortisol down regulate each other (56, 57), the steady decrease of cortisol might be due to the intense and prolonged increase of testosterone rather than a semiochemical effect elicited by periovulatory vulva odors. Cortisol peaked 30 min after smelling periovulatory axilla odors, subsequently returning to basal levels. Cortisol decreased significantly 15 and 60 min after smelling luteal axilla odors, strengthening the idea that luteal odors act as a stimulus that reduces overall arousal. A great peak of cortisol occurred 30 min after smelling luteal vulva odors. The effect elicited by luteal vulva scents are similar to that elicited by a stressful social situation (44) suggests these odors are perceived as aversive.

According to our predictions only periovulatory scents increased interest in sex, while luteal odors did not. However, ours was a small questionnaire implemented to gain insight on sexual thoughts, and the results, though significant are modest. Perhaps we applied this questionnaire too far away from the odors stimuli, or the neutral videos participants had to watched waned interest in sex. Further studies using a structured, reliable, and specifically designed questionnaire, such as the sexual desire inventory (58) might reveal more interesting results. Nonetheless, taking in account the significance levels and the effect sizes, the periovulatory axilla odors were somewhat more powerful than the vulvar scents, despite the sustained increase of testosterone facilitated by the latter. As such, axillary odors stand out as a more natural source of chemical communication than genital odors in humans.

In common with other studies (4, 20–22), periovulatory scents from the axilla and the vulva were considered significantly more pleasant, but not more intense, than air. That luteal axilla scents were not perceived as intense confirms Havlícek et al. (4, 41) results that intensity does not vary between the fertile and non-fertile period and that intensity is low when sampling odors for short periods (≤12 h). The luteal vulva odor was rated as unpleasant and highly intense, while eliciting a great cortisol peak 30 min after smelling these scents. Finally, axillary and vulvar scents collected in the periovulatory period and particularly the luteal vulva odors were rated as significantly more familiar than air. We cannot be sure if such appreciation, especially for the vulvar scents, came from our participants’ sexual experience. A more parsimonious explanation could be that they recognized sweat in the armpit and the fairly common odors of certain aliphatic acids such as butyric, propionic, and acetic acids, and broke-down amino acids (in particular regarding luteal vulva effects) found in the female genitals.

Our view is that aliphatic acids and other small molecules do not stimulate sexual arousal, because they are real odorants consciously recognized and are found in a wide variety of products such as vinegar, fruits, etc. Only by association with pleasurable sexual experiences this compounds could increase sexual arousal. On the other hand, recent research has found that odorless steroid metabolites account for pheromonal-like effects [e.g., Ref. (9) and references within]. Thus, it is possible that conscious perception of known odorants found in the axilla and vulva, coupled with the psychoneuroendocrinological effects of odorless “pheromones” jointly contribute to enhance sexual arousal. Which leads to our final conclusion.

Our study was not aimed to investigate the relationship of vulvar scents and cunnilingus. However, the sustained increase in testosterone and the concomitant (though perhaps incidental) decrease in cortisol elicited by periovulatory scents supports Pham’s and Shackelford’ (30) claim that cunnilingus promotes future copulation. Our results indicate cunnilingus could facilitate further copulations in a same intercourse session, since besides from heightening sexual energy, high testosterone levels might motivate interest in and caressing of the couple (32), while low cortisol levels might promote intimacy (59).

## Authors Contribution

All authors contributed equally to the formulation and design of this study. Ana Lilia Cerda-Molina and Leonor Hernández-López attended the female participants and did the hormonal extractions from saliva. Roberto Chavira-Rodríguez did the immunoanalyses, Claudio Eric de la O built the database, and Ricardo Mondragón-Ceballos did the statistical analyses. All authors participated in drafts’ corrections, while Ana Lilia Cerda-Molina, Leonor Hernándes-López, and Ricardo Mondragón-Ceballos wrote the final version of the paper.

## Conflict of Interest Statement

The authors declare that the research was conducted in the absence of any commercial or financial relationships that could be construed as a potential conflict of interest.
